# Taxonomic revision of the Nippostrongylinae (Nematoda, Heligmonellidae) parasites of Muridae from the Australasian region. The genus *Odilia* Durette-Desset, 1973

**DOI:** 10.1051/parasite/2015032

**Published:** 2015-11-23

**Authors:** Marie-Claude Durette-Desset, María Celina Digiani

**Affiliations:** 1 ISYEB, Institut Systématique, Évolution, Biodiversité, UMR7205 CNRS, EPHE, MNHN, UPMC, Muséum National d’Histoire Naturelle, Sorbonne Universités 61 rue Buffon 75231 Paris Cedex 05 France; 2 CONICET-Consejo Nacional de Investigaciones Científicas y Técnicas Argentina; 3 División Zoología Invertebrados, Facultad de Ciencias Naturales y Museo, Universidad Nacional de La Plata, Paseo del Bosque s/n 1900 La Plata Argentina

**Keywords:** Trichostrongylina, Synlophe, New genera, Australia, Indonesia, New Guinea

## Abstract

The species of the genus *Odilia* Durette-Desset, 1973 (Heligmonellidae, Nippostrongylinae) are re-distributed among eight genera of which five are new. This classification is mainly based on certain characters of the synlophe not previously taken into account at the supraspecific level. These characters mainly include the presence or absence of a careen, the relative size of the ridges forming the careen, the development and position of ridge 1’, the development of the left ridge and right ridge, and the distribution of the largest ridges. Eighteen of the 20 known species are rearranged in the following genera: *Odilia sensu stricto* Durette-Desset, 1973 with *Odilia mackerrasae* (Mawson, 1961) as type species, *Chisholmia* n. gen. with *Chisholmia bainae* (Beveridge & Durette-Desset, 1992) n. comb. as type species, *Equilophos* n. gen. with *Equilophos polyrhabdote* (Mawson, 1961) n. comb. as type species, *Hasegawanema* n. gen. with *Hasegawanema mamasaense* (Hasegawa, Miyata & Syafruddin, 1999) n. comb. as type species, *Hughjonestrongylus* Digiani & Durette-Desset, 2014 with *Hughjonestrongylus ennisae* (Smales & Heinrich, 2010) as type species, *Lesleyella* n. gen. with *Lesleyella wauensis* (Smales, 2010) n. comb. as type and sole species, *Parasabanema szalayi* Smales & Heinrich, 2010, and *Sanduanensis* n. gen. with *Sanduanensis dividua* (Smales, 2010) as type and sole species. *Odilia uromyos* Mawson, 1961 and *Odilia carinatae* Smales, 2008 are not included in the new classification. A key to the proposed genera is provided. The new generic arrangement follows a distribution more related to the biogeographical areas than to the host groups.

## Abstract (fr)

Les espèces du genre *Odilia* Durette-Desset, 1973 (Heligmonellidae, Nippostrongylinae) sont réparties en huit genres, dont cinq nouveaux. Cette classification s’appuie principalement sur certains caractères du synlophe non pris en compte jusqu’à présent au niveau supra-spécifique. Ces caractères incluent principalement la présence ou l’absence d’une carène, la taille relative des crêtes formant la carène, le développement et la position de la crête 1’, le développement de la crête gauche et de la crête droite, et la répartition des crêtes les plus larges. Dix-huit espèces sur les 20 actuellement connues sont classées dans les genres suivants: *Odilia sensu stricto* Durette-Desset, 1973 avec *Odilia mackerrasae* (Mawson, 1961) comme espèce type, *Chisholmia* n. gen. avec *Chisholmia bainae* (Beveridge & Durette-Desset, 1992) n. comb. comme espèce type, *Equilophos* n. gen. avec *Equilophos polyrhabdote* (Mawson, 1961) n. comb. comme espèce type, *Hasegawanema* n. gen. avec *Hasegawanema mamasaense* (Hasegawa, Miyata & Syafruddin, 1999) n. comb. comme espèce type, *Hughjonestrongylus* Digiani & Durette-Desset, 2014 avec *Hughjonestrongylus ennisae* (Smales & Heinrich, 2010) comme espèce type, *Lesleyella* n. gen. avec *Lesleyella wauensis* (Smales, 2010) n. comb. comme espèce type et unique espèce, *Parasabanema szalayi* Smales & Heinrich, 2010 et *Sanduanensis* n. gen. avec *Sanduanensis dividua* (Smales, 2010) comme espèce type et unique espèce. *Odilia uromyos* Mawson, 1961 et *Odilia carinatae* Smales, 2008 ne sont pas incluses dans la nouvelle classification. Une clé dichotomique des genres proposés est fournie. Le nouvel arrangement générique suit une répartition plus liée aux aires biogéographiques qu’aux groupes d’hôtes.

## Introduction

This paper contains a taxonomic revision of the Australasian Nippostrongylinae. In a preceding paper, we studied the *Paraheligmonelloides* complex, which was divided into four genera [4]. The present paper concerns the genus *Odilia* Durette-Desset, 1973, with 20 described species in murids from mainland Australia, Tasmania, Borneo, Sulawesi, and New Guinea.

Durette-Desset [6] created the nematode genus *Austrostrongylus* to include the following species parasitic in Australian Muridae: *Heligmonoides mackerrasae* Mawson, 1961 (type species), *Longistriata brachybursa* Mawson, 1961, *Heligmonoides emanuelae* Mawson, 1961, *Heligmonoides mawsonae* Durette-Desset, 1969, *Longistriata melomyos* Mawson, 1961, *Longistriata polyrhabdote* Mawson, 1961, and *Longistriata uromyos* Mawson, 1961 [17]. As the name *Austrostrongylus* was preoccupied, created by Chandler [2] for nematode parasites of Australian marsupials, with *A. macropodis* as type species, Durette-Desset [7] proposed the new name *Odilia* for the parasites of Australian Muridae. Probably by omission, the species *brachybursa* was not included in the new combinations with *Odilia.*

The synlophe of these species (except *O. uromyos*) was described by Durette-Desset [5] and the genus *Odilia* (=*Austrostrongylus sensu* Durette-Desset, 1971, *nec* Chandler, 1924) was defined as follows: “synlophe with axis of orientation directed from right-ventral line to left-dorsal line; hypertrophied lateral ridges; left-dorsal ridge almost as long as left-ventral ridge tending to the formation of a small careen, the latter remaining of moderate size. Gradient in ridge size latero-median or ridges very numerous and of similar size” [6].

After a gap of approximately 20 years, eight new species were described between 1992 and 2005: three from mainland Australia and Tasmania [1, 11], and five from Indonesia [12, 16]. Smales [20] proposed a key to the 15 species then known in the genus, mainly based on the number of cuticular ridges and, to a lesser extent, on the length of the gubernaculum. Since 2005, five other species from New Guinea have been described and assigned to *Odilia* [21–23, 25], bringing the number of species in the genus to 20.

At present, the composition of the genus is very heterogeneous, mainly due to the great variability of the synlophe among the species: some species possess a careen, whereas other species do not, the number of ridges varies from 14 to 35, the lateral ridges are not always well developed, and the ridge size also varies considerably.

It is likely, in one way, that the generic definition was sufficiently ambiguous to allow the inclusion of species with very different synlophes. In another way, however, some species do not even match the generic definition.

In an ongoing revision of the Heligmonellidae, it became necessary to review the specific composition of *Odilia*, and to attempt to group the species into new, possibly supraspecific taxa. This work is presented here, based mainly on the morphology of the synlophe, a complex structure involving numerous characters, some of which are proving to be taxonomically useful at the supraspecific level.

## Materials and methods

The data were compiled from the published descriptions. The species whose synlophe could be analyzed were: *Odilia mackerrasae* (Mawson, 1961), *Odilia bainae* Beveridge & Durette-Desset, 1992, *Odilia brachybursa* (Mawson, 1961), *Odilia carinatae* Smales, 2008, *Odilia dividua* Smales, 2014, *Odilia emanuelae* (Mawson, 1961), *Odilia implexa* Smales, 2008, *Odilia mallomyos* Hasegawa & Syafruddin, 1994, *Odilia mamasaensis* Hasegawa, Miyata & Syafruddin, 1999, *Odilia mawsonae* (Durette-Desset, 1969), *Odilia maxomyos* Hasegawa, Miyata & Syafruddin, 1999, *Odilia melomyos* (Mawson, 1961), *Odilia moatensis* Hasegawa, Miyata & Syafruddin, 1999, *Odilia polyrhabdote* (Mawson, 1961), *Odilia praeputialis* Gibbons & Spratt, 1995, *Odilia similis* Smales, 2009, *Odilia tasmaniensis* Gibbons & Spratt, 1995, *Odilia sulawesiensis* Hasegawa, Miyata & Syafruddin, 1999, and *Odilia wauensis* Smales, 2010. The species *Odilia uromyos* (Mawson, 1961) as well as *Odilia* sp. 1 and *Odilia* sp. 2 of Hasegawa & Syafruddin [14], whose synlophes were not illustrated, were not included in the study.

**Figure 1.**
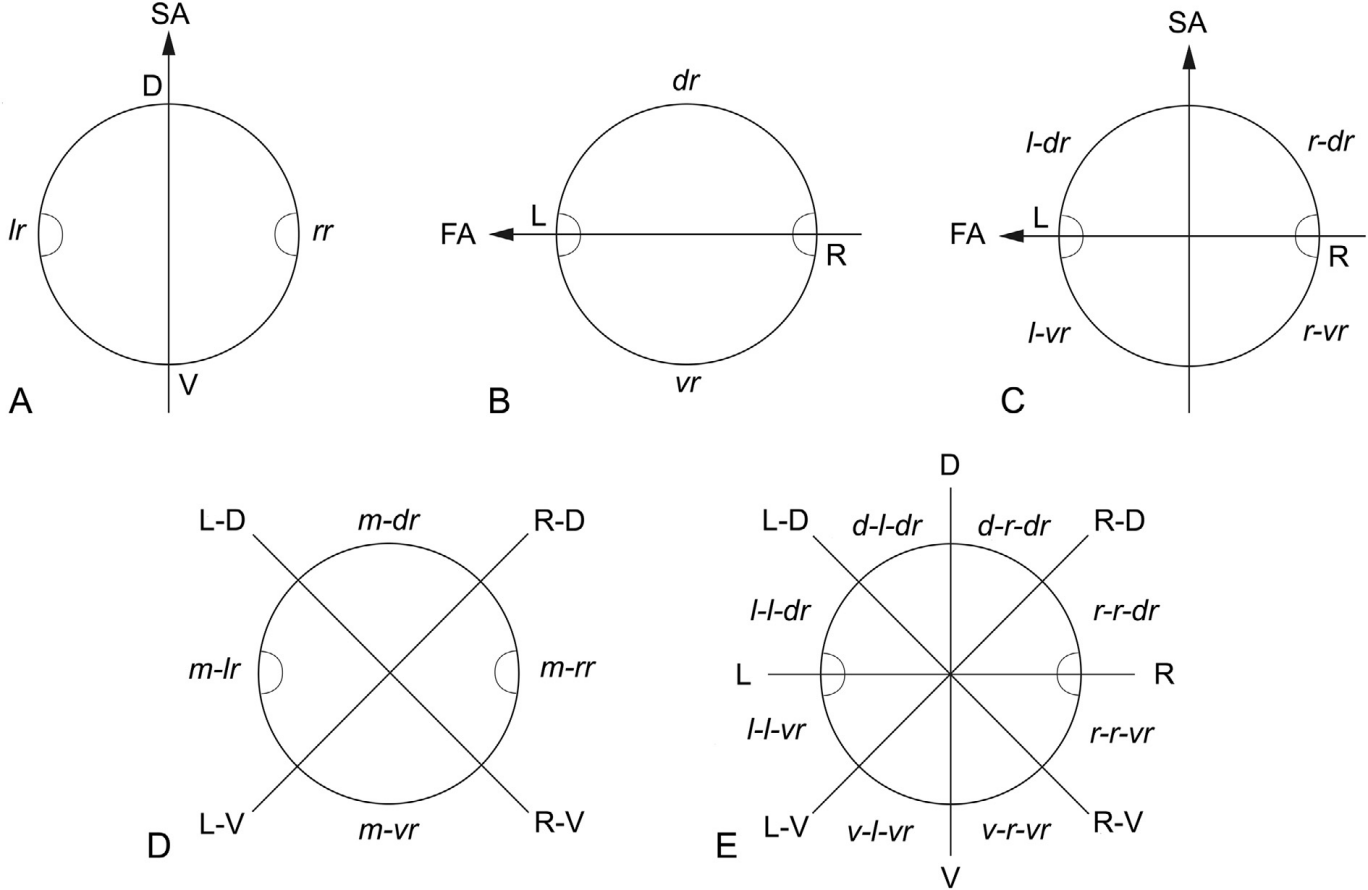
(A–E) Distribution of ridges around the body circumference. (A) According to the sagittal axis, ridges are named left ridges and right ridges. (B) According to the frontal axis, ridges are named dorsal ridges and ventral ridges. (C) Division into quadrants resulting from the intersection of sagittal and frontal axes. Ridges are named left-dorsal, right-dorsal, right-ventral, and left-ventral ridges. (D) Division into quadrants resulting from the intersection of the axes “right-ventral/left-dorsal” and “left-ventral/right-dorsal”. Ridges are named mid-dorsal, mid-right, mid-ventral, and mid-left ridges. (E) Division into octants. Ridges are named dorsal-right-dorsal, right-right-dorsal, right-right-ventral, ventral-right-ventral, ventral-left-ventral, left-left-ventral, left-left-dorsal, and dorsal-left-dorsal ridges. *Abbreviations*: D: dorsal side; FA: frontal axis; L: left side; LV/RD: left-ventral/right-dorsal axis; R: right side; RV/LD: right-ventral/left-dorsal axis; SA: sagittal axis; V: ventral side; dr: dorsal ridges; d-l-dr: dorsal-left-dorsal ridges; d-r-dr: dorsal-right-dorsal ridges; lr: left ridges; l-dr: left-dorsal ridges; l-vr: left-ventral ridges; l-l-dr: left-left-dorsal ridges; l-l-vr: left-left-ventral ridges; m-dr: mid-dorsal ridges; m-lr: mid-left ridges, m-rr: mid-right ridges, m-vr: mid-ventral ridges; r-dr: right-dorsal ridges; rr: right ridges; r-vr: right-ventral ridges; r-r-dr: right-right-dorsal ridges; r-r-vr: right-right-ventral ridges; vr: ventral ridges, v-l-vr: ventral-left-ventral ridges; v-r-vr: ventral-right-ventral ridges.

The methods used for the study and description of the synlophe follow the terms and criteria provided by Durette-Desset [8] and Durette-Desset & Digiani [9]. To indicate more accurately the position of the ridges around the body circumference, the body section may be divided primarily into hemispheres or sides: right side and left side determined by the sagittal axis (SA) (Fig. 1A), and dorsal side and ventral side determined by the frontal axis (passing through the lateral hypodermal cords) (FA) (Fig. 1B). Applying the same principle, the ridges situated on the dorsal side are named dorsal ridges, those on the left side left ridges, etc. More complex synlophes usually require for their description a division of the section into quadrants or even into octants. The intersection of the SA and FA determines four quadrants referred as to left-dorsal, right-dorsal, right-ventral, and left-ventral (Fig. 1C). Similarly, the intersection of the diagonals of the quadrants defined above determines four other quadrants which may be referred to as mid-dorsal, mid-right, mid-ventral, and mid-left (Fig. 1D). The ridges situated in the different quadrants are named following the same principle as right-ventral ridges, left-dorsal ridges, etc. (Figs. 1C and 1D). A division into octants results in sections identified as dorsal-right-dorsal, right-right-dorsal, right-right-ventral, ventral-right-ventral, ventral-left-ventral, left-left-ventral, left-left-dorsal, and dorsal-left-dorsal, and the ridges on them are named consequently as dorsal-right-dorsal ridges, right-right-dorsal ridges, etc. (Fig. 1E).

As proposed in a previous article [4], the terms “right ridge” and “left ridge” when used in the singular, indicate the single ridge closest to the right and left lateral fields, respectively.

**Figure 2.**
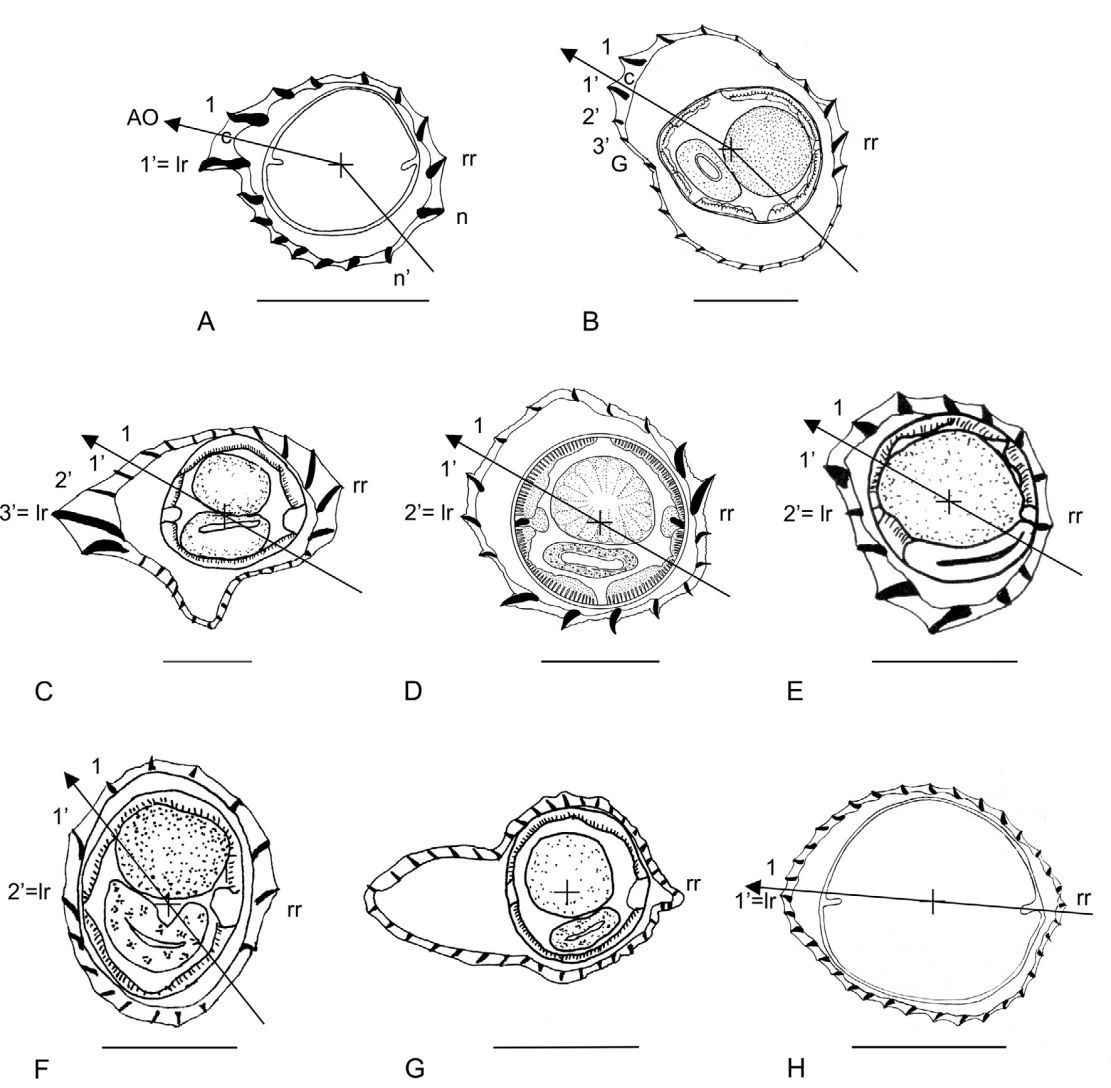
Synlophes at mid-body of the genera treated in this work. Type-species. (A) *Odilia* (*O. mackerrasae*), male. (B) *Hasegawanema* n. gen. (*Hasegawanema mamasaense* n. comb), female. C, *Hughjonestrongylus* (*H. ennisae*), female. (D) *Chisholmia* n. gen. (*Chisholmia bainae* n. comb.), male. (E) *Lesleyella* n. gen. (*Lesleyella wauensis* n. comb.), female. (F) *Sanduanensis* n. gen. (*Sanduanensis dividua* n. comb.), female. (G) *Parasabanema* (*P. szalayi*), male. (H) *Equilophos* n. gen. (*Equilophos polyrhabdote* n. comb.), female. *Abbreviations*: 1, 1’, 2’, 3’: ridges 1, 1’, 2’, 3’; AO: axis of orientation of the ridges; c: careen; G: gap; lr: left ridge; n: last dorsal ridge; n’: last ventral ridge; rr: right ridge. A, H, modified from [5]. B, modified from [16]. C, G, modified from [26]. D, modified from [1]. E, modified from [20]. F, modified from [25]. *Scale-bars*: 50 mm.

In the Heligmosomoidea, the ridges are usually numbered according to an axis of orientation which separates them into two groups with tips pointing in opposing directions. The axis of orientation is always directed from the right-ventral quadrant to the left-dorsal quadrant and in the Nippostrongylinae, its inclination ranges from 1° to 90° on the sagittal axis, depending on the species. At 90°, it overlaps the frontal axis passing through the lateral cords. The numbering of the ridges always begins on the left side and by definition, ridge 1 is situated dorsally to the axis and ridge 1’ is situated ventrally. If the axis is frontal, it separates one group of dorsal ridges numbered 1 to *n* and another of ventral ridges numbered 1’ to *n*’. If the axis is oblique, it separates one group of right-dorsal ridges numbered 1 to *n* and another of left-ventral ridges numbered 1’ to *n*’ (Fig. 2A).

The left ridge may be homologous with ridge 1’ (Fig. 2A) or distinct from it (Figs. 2B–G). This seems to be a strong character which allowed the rearrangement of several species in the revision of genera such as in *Neoheligmonella* Durette-Desset, 1971 [3] and *Paraheligmonelloides* Fukumoto, Kamiya & Suzuki, 1980 [4].

The main synlophe characters used to separate the different genera were the following: (1) presence or absence of a careen; (2) size of ridges forming the careen; (3) position and development of ridge 1’; (4) development of the left ridge; (5) development of the right ridge; (6) size of the ridges; (7) distribution of the largest ridges; (8) presence of cuticular dilatations; and (9) discontinuity of ridges.

The description of the bursa follows Durette-Desset & Digiani [10]. Other characters, especially of the bursa and spicules are included in the generic definitions. The abbreviation SpL/BL refers to the spicule length as a proportion of the body length, expressed as a percentage. The nomenclature of the hosts and their taxonomy at the suprageneric level follow Musser & Carleton [18].

## Results

### Characters of the Synlophe

1. Presence or absence of a careen and
2. relative size of ridges forming the careen
  - The species *O. brachybursa, O. emanuelae, O. mackerrasae, O. melomyos,* and *O. tasmaniensis* possess a careen made up of two well-developed ridges, larger than the others. The ventral ridge of the careen is slightly larger than the dorsal one in *O. brachybursa, O. mackerrasae,* and *O. tasmaniensis*; markedly larger in *O. emanuelae* and *O. melomyos.*
  - The species *O. mallomyos*, *O. mamasaensis*, *O. maxomyos*, *O. moatensis*, and *O. sulawesiensis* possess a careen made up of two thin ridges, comparable in size to the right ridge and the ridges adjacent to it. Both ridges of the careen have the same size in *O. mamasaensis*, *O. moatensis*, and *O. sulawesiensis*; the ventral ridge is slightly larger in *O. mallomyos* and *O. maxomyos*.
  - The careen is absent in *O. bainae, O. carinatae, O. dividua*, *O. implexa, O. mawsonae, O. polyrhabdote, O. praeputialis, O. similis,* and *O. wauensis*.
3. Position and development of ridge 1’
  - Ridge 1’ is the left ridge, situated in the left lateral field.
    - It is the largest ridge in *O. brachybursa, O. emanuelae*, *O. mackerrasae, O. melomyos*, and *O. tasmaniensis*.
    - It is as developed as the other ridges in *O. mawsonae, O. polyrhabdote,* and *O. similis.*
  - Ridge 1’ is not the left ridge and is situated in the dorsal left quadrant.
  - Ridge 1’ is among the largest ridges in *O. carinatae*, *O. dividua*, *O. mallomyos*, *O. mamasaensis*, *O. maxomyos*, *O. moatensis*, *O. sulawesiensis*, and *O. wauensis.*
  - Ridge 1’ is among the smallest ridges in *O. bainae*, *O. mawsonae,* and *O. implexa*.
  - Ridge 1’ is as developed as the other left ridges in *O. praeputialis.*
4. Development of the left ridge
  - The left ridge is the largest ridge in *O. brachybursa, O. emanuelae*, *O. mackerrasae*, *O. melomyos*, and *O. tasmaniensis* (where it is homologous with ridge 1’).
  - It is among the largest ridges in *O. dividua* and *O implexa*.
  - It is among the smallest ridges in *O. bainae*, *O. carinatae*, *O. mawsonae*, and *O. wauensis*.
  - It is small and similar in size to all the other ridges in *O. polyrhabdote* and *O. similis*.
  - It is as small as the other left ridges in *O. praeputialis.*
  - It is replaced by a gap in *O. mallomyos, O. mamasaensis, O. maxomyos, O. moatensis*, and *O. sulawesiensis.*
5. Development of the right ridge
  - The right ridge is well developed and larger than the adjacent ridges in *O. bainae*, *O. brachybursa, O. carinatae*, *O. emanuelae*, *O. mackerrasae, O. mallomyos, O. mamasaensis*, *O. mawsonae*, *O. maxomyos, O. melomyos*, *O. moatensis*, *O. tasmaniensis*, and *O. sulawesiensis.*
  - It is well developed and of comparable size to the adjacent ridges in *O. implexa*.
  - It is moderately developed and of the same size as the adjacent ridges in *O. carinatae, O. dividua* and *O. mackerrasae.*
  - It is poorly developed and smaller than the right-dorsal ridges but larger than the right-ventral ridges in *O. praeputialis* and *O. wauensis*.
  - It is poorly developed and of similar size to the remaining ridges in *O. polyrhabdote* and *O. similis*.
6. Size of the ridges
  - All ridges are small and of similar size in *O. polyrhabdote*, *O. similis*, and *O. praeputialis*.
  - Ridges are unequal in size, but not markedly. Medium-sized to small ridges in *O. bainae*, *O. brachybursa*, *O. carinatae*, *O. dividua*, *O. emanuelae*, *O. mallomyos*, *O. mackerrasae*, *O. mawsonae, O. melomyos*, *O. tasmaniensis* and *O. wauensis.* Small to minute ridges in *O. mamasaensis*, *O. maxomyos*, *O. moatensis,* and *O. sulawesiensis.*
  - Ridges are markedly unequal in size (few hypertrophied ridges and remaining ridges small) in *O. implexa.*
  No clear gradients in ridge size were observed in the species studied. In certain species, such as *O. mackerrasae*, *O. tasmaniensis*, *O. maxomyos*, and *O. implexa,* diminishing gradients in size were observed but frequently in only one of both sexes and it is apparently not a stable character.
7. Position of the largest ridges
  - In *O. mackerrasae*, *O. mallomyos, O. mamasaensis*, *O. maxomyos*, *O. moatensis (female)*, and *O. sulawesiensis* the largest ridges are the careen and the ridges associated with the right ridge (right ridge plus 1–2 ridges situated dorsally to it).
  - In *O. brachybursa*, *O. emanuelae*, *O. melomyos*, and *O. tasmaniensis* the largest ridges are the careen, the ridges associated with the right ridge, and the ventral-left-ventral ridges.
  - In *O. wauensis* the largest ridges are ridge 1’, and the ventral-left-ventral ridges.
  - In *O. implexa, O. carinatae* females, and *O. dividua* the largest ridges are the mid-left and mid-right ridges.
  - In the male of *O. carinatae* the largest ridges are the careen and the mid-right ridges.
  - In *O. bainae* the largest ridges are the ridges associated with the right ridge and the ventral-left-ventral ridges.
  - In the male of *O. mawsonae*, at mid-body, the largest ridges are the ridges associated with the right ridge. In the anterior part of the body, the largest ridges are also the mid-left and left-ventral ridges.
  - In *O. polyrhabdote* and *O. similis* no ridges are markedly larger than the others.
  - In *O. praeputialis* the largest ridges are the mid-left ridges, and dorsal, right-dorsal ones.
8. Presence of cuticular dilatations
  - Two cuticular dilatations (or at least one), situated in the left-dorsal and right-ventral quadrants, are present in *O. brachybursa*, *O. carinatae*, *O. emanuelae*, *O. implexa*, *O. mackerrasae*, *O. mallomyos*, *O. mamasaensis*, *O. mawsonae*, *O. maxyomyos*, *O. melomyos*, *O. moatensis*, *O. praeputialis*, *O. sulawesiensis,* and *O. tasmaniensis.*
  - The cuticular dilatations are absent in *O. bainae*, *O. dividua*, *O. mawsonae*, *O. polyrhabdote*, *O. similis*, and *O. wauensis.*
9. Discontinuity of ridges
  - In *O. mackerrasae* and *O. dividua* the ridges are discontinuous on the ventral side of the body. They are continuous in the other species.

## Discussion

Since some of the characters analyzed were frequently associated with one another, we were able to group the species treated into eight groups:

### Groups (1 and 2), species with a careen

1. Species with a careen of medium size and right ridge moderately developed. The left ridge is ridge 1’. The largest ridges are the careen, the ridges associated with the right ridge and the left-ventral ridges (though in *O. mackerrasae* the left-ventral ridges are small). Presence of double cuticular dilatation. Five species of *Odilia*: *O. mackerrasae* (type species of the genus), *O. brachybursa*, *O. emanuelae*, *O. melomyos*, and *O. tasmaniensis* (Fig. 2A).
2. Species with a careen of small size and right ridge comparable in size to careen. Ridges adjacent to careen very small. Left ridge distinct from ridge 1’, poorly developed or replaced by a gap. Though small, the careen and the right ridge are the largest ridges. Presence of double cuticular dilatation. Five species: *O. mallomyos*, *O. mamasaensis*, *O. maxomyos*, *O. moatensis* (female), and *O. sulawesiensis* (Fig. 2B, 3B–E).
  **Figure 3.**
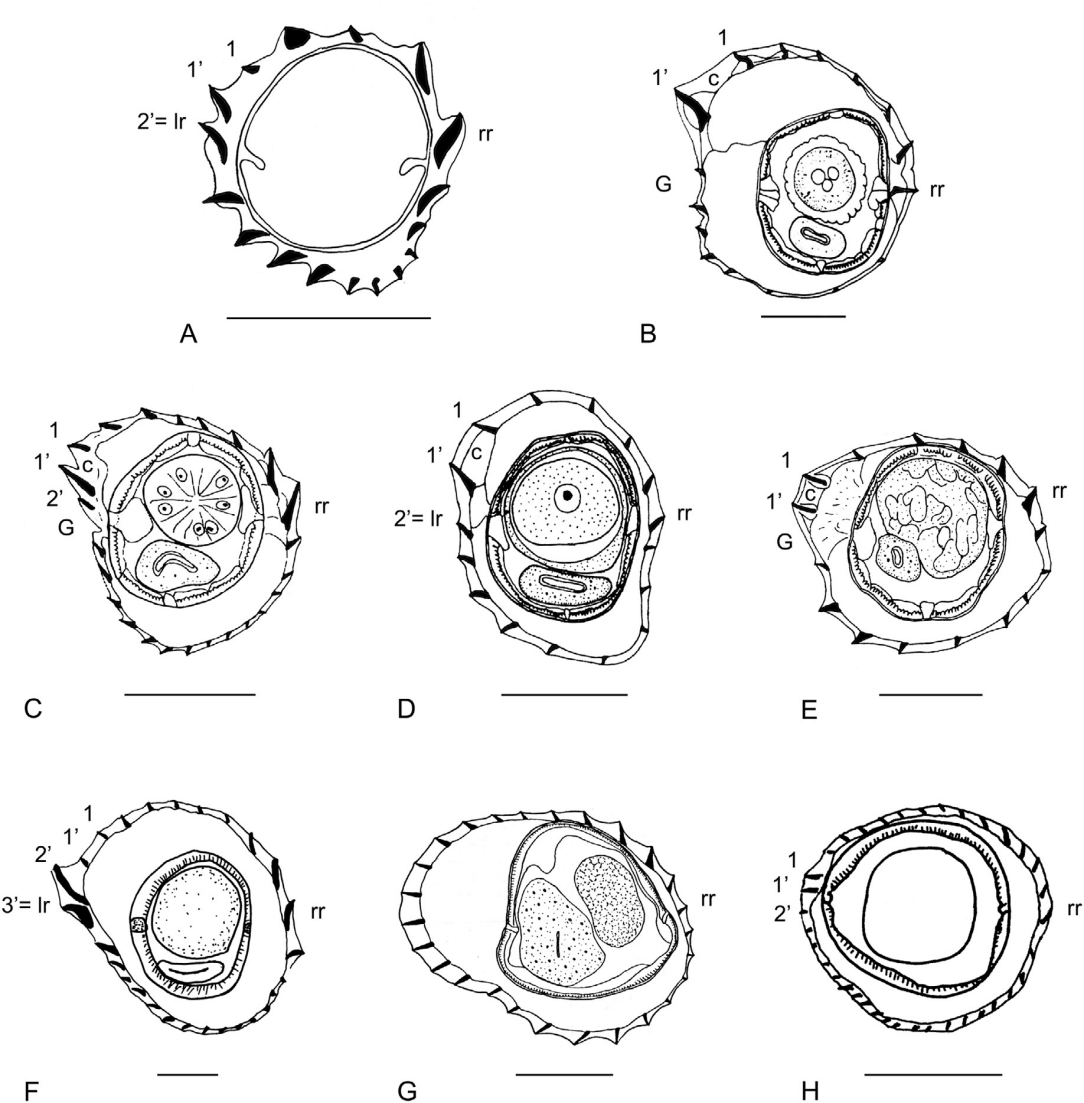
Synlophes of the genera treated in this work. Other species. (A) within anterior part of body, *Chisholmia mawsonae* n. comb., male. (B–H) at mid-body: (B) *Hasegawanema mallomyos* n. comb., female. (C) *Hasegawanema maxomyos* n. comb., male. (D) *Hasegawanema moatense* n. comb., female. (E) *Hasegawanema sulawesiense* n. comb., female. (F) *Hughjonestrongylus implexus* n. comb., female. (G) *Parasabanema praeputiale* n. comb., female. (H) *Equilophos similis* n. comb., male. *Abbreviations*: 1, 1’, 2’, 3’: ridges 1, 1’, 2’, 3’; c: careen; G: gap; lr: left ridge; rr: right ridge. A, modified from [5]. B, modified from [12]. C–E, modified from [16]. F, modified from [21]. G, modified from [11]. H, modified from [22]. *Scale-bars*: 50 mm.

### Groups (3–8), species without a careen

1. Species with ridges markedly unequal in size. Left ridge distinct from ridge 1’. Mid-left and mid-right ridges largest. Presence of double cuticular dilatation. One species: *O. implexa* (Fig. 3F).
2. Species with ridges slightly unequal in size. Left ridge distinct from ridge 1’. The largest ridges are those associated with the right ridge and the left-ventral ridges. Cuticular dilatations absent. Two species: *O. bainae* (Fig. 2D) and *O. mawsonae* (Fig. 3A).
3. Species with ridges unequal in size. Left ridge distinct from ridge 1’. Left ridge medium-sized, right ridge small. Largest ridges: ridge 1’, right-right-dorsal ridges (except the right ridge) and ventral-left-ventral ridges. Cuticular dilatations absent. One species: *O. wauensis* (Fig. 2E).
4. Species with ridges slightly unequal in size. Left ridge distinct from ridge 1’. Left ridge and right ridge of similar size. Mid-left ridges and mid-right ridges largest. Cuticular dilatations absent. One species: *O. dividua* (Fig. 2F).
5. Species with small ridges slightly unequal in size. Left ridges largest. Presence of double cuticular dilatation. One species: *O. praeputialis* (Fig. 3G).
6. Species with small ridges subequal in size. Mid-right ridges minute. Cuticular dilatations absent. Two species: *O. polyrhabdote* (Fig. 2H) and *O. similis* (Fig. 3H).

## New classification proposed

Based on the groups of species mentioned above, we propose to group the species of the present-day genus *Odilia* into eight genera of which five are new. Seven out of the 18 species studied are attributed to the existing genera *Odilia sensu stricto* Durette-Desset, 1973 (5 species), *Hughjonestrongylus* Digiani & Durette-Desset, 2014 (1 species), and *Parasabanema* Smales & Heinrich, 2010 (1 species). The other species were distributed in the following new genera: *Hasegawanema* n. gen. (5 species), *Chisholmia* n. gen. (2 species), *Lesleyella* n. gen. (1 species), *Sanduanensis* n. gen. (1 species), and *Equilophos* n. gen. (2 species).

### I- Genus *Odilia* Durette-Desset, 1973 (Fig. 2A)

Type species: *Odilia mackerrasae* (Mawson, 1961).

Hosts: Muridae (Rodentia).

Host site: Small intestine.

Distribution: Mainland Australia, Tasmania.

Definition: Heligmonellidae, Nippostrongylinae. Synlophe with 14–18 ridges in both sexes. Ridges continuous (except type species with ventral ridges discontinuous). Careen supported by two medium-sized ridges, at least in proximal part of body, with ventral ridge longer. Ridge 1’ is the left ridge. Ridges unequal in size, median to small. Careen, ridges associated with the right ridge, and ventral-left-ventral ridges, largest. Other ridges (mid-dorsal, right-ventral) smaller. Presence of two minute ridges or a gap dorsally adjacent to largest left-ventral ridges (except type species). Cuticular dilatations situated on left-dorsal and right-ventral quadrants. Axis (es) of orientation oblique. Characteristic bursal pattern of types 2-2-1, 1-3-1. Dorsal ray divided within proximal half. Each spicule ending in one tip. SpL/BL: 7–16%.

Other species: *O. brachybursa* (Mawson, 1961), *O. emanuelae* (Mawson, 1961), *O. melomyos* (Mawson, 1961), *O. tasmaniensis* Gibbons & Spratt, 1995.

### II- Genus *Hasegawanema* n. gen. (Figs. 2B and 3B-E)

urn:lsid:zoobank.org:act:A3C86F23-57EA-4BE5-889E-377490B752CB

Type species: *Hasegawanema mamasaense* (Hasegawa, Miyata & Syafruddin, 1999) n. comb.

Hosts: Muridae (Rodentia).

Host site: Small intestine.

Distribution: Indonesia.

Etymology: The genus is named in honor of Pr. Hideo Hasegawa (Faculty of Medicine, Oita University, Japan), in recognition of his valuable contribution to knowledge on the Oriental Nippostrongylinae.

Definition: Heligmonellidae, Nippostrongylinae. Synlophe with 15–26 ridges in both sexes. Ridges continuous. Careen supported by two small ridges with ventral one generally slightly larger. Ridge 1’ distinct from left ridge. Ridges unequal in size, small to minute. Careen and ridges associated with right ridge, largest. Other ridges very small or minute. Left ridge minute or replaced by a gap in front of the left lateral field. Axis (es) of orientation oblique. Characteristic bursal pattern of type 2-2-1. Dorsal ray divided within proximal half. Each spicule ending in one tip. SpL/BL: 6–25%.

Other species: *Hasegawanema mallomyos* (Hasegawa & Syafruddin, 1994) n. comb., *Hasegawanema maxomyos* (Hasegawa, Miyata & Syafruddin, 1999) n. comb., *Hasegawanema moatense* (Hasegawa, Miyata & Syafruddin, 1999) n. comb., *Hasegawanema sulawesiense* (Hasegawa, Miyata & Syafruddin, 1999) n. comb.

### III- Genus *Hughjonestrongylus* Digiani & Durette-Desset, 2014 (Figs. 2C and 3F)

Type species: *Hughjonestrongylus ennisae* (Smales & Heinrich, 2010) Digiani & Durette-Desset, 2014.

Hosts: Muridae (Murinae).

Host site: Small intestine.

Distribution: Papua New Guinea, Papua Indonesia.

Definition: Heligmonellidae, Nippostrongylinae. Synlophe with 20–30 ridges in both sexes. Ridges continuous. Careen absent. Ridges markedly unequal in size. Left ridge distinct from ridge 1’. Mid-left and mid-right ridges largest. Left ridges generally larger than right ones. Presence of left-dorsal and right-ventral cuticular dilatations. Axis(es) of orientation oblique. Characteristic bursal pattern of types 1–4 and 2–3. Dorsal ray divided within distal half. Spicules thick, each one ending in one or three tips. SpL/BL 10–15%.

Other species: *Hughjonestrongylus amplicaudae* (Smales & Heinrich, 2010) Digiani & Durette-Desset, 2014, *Hughjonestrongylus implexus* (Smales, 2008) n. comb., *Hughjonestrongylus mirzai* (Smales, 2009) Digiani & Durette-Desset, 2014, *Hughjonestrongylus singauwaensis* (Smales & Heinrich, 2010) Digiani & Durette-Desset, 2014, *Hughjonestrongylus*. sp. of Smales [24].

### IV- Genus *Chisholmia* n. gen. (Figs. 2D and 3A)

urn:lsid:zoobank.org:act:680A783E-5BB1-473A-836F-4E6DB762F649

Type species: *Chisholmia bainae* (Beveridge & Durette-Desset, 1992) n. comb.

Hosts: Muridae (Murinae).

Host site: Small intestine.

Distribution: Mainland Australia, Tasmania.

Etymology: The genus is named in honor of Dr. Leslie Chisholm, recognized researcher on marine parasites at the University of Adelaide (Australia), and Manager of the Parasitology and Arachnology Collections at the South Australian Museum.

Definition: Heligmonellidae, Nippostrongylinae. Synlophe with 16–22 ridges in both sexes. Ridges continuous. Careen absent. At least in proximal part of body, ridges slightly unequal in size, median to small. Left ridge distinct from ridge 1’. Left ridge smaller than right ridge. Ridges associated with right ridge and ventral-left ridges largest. Axis (es) of orientation oblique. Absence of cuticular dilatations. Characteristic bursal pattern of types 2-2-1 and 1–4. Dorsal ray divided within proximal half. Each spicule ending in one tip. SpL/BL: 7–13%.

Other species: *Chisholmia mawsonae* (Durette-Desset, 1969) n. comb.

### V- Genus *Lesleyella* n. gen. (Fig. 2E)

urn:lsid:zoobank.org:act:241C45B2-8EE9-419E-B14B-7A85990D2C3D

Type and sole species: *Lesleyella wauensis* (Smales, 2010) n. comb.

Hosts: Muridae (Rodentia).

Host site: Small intestine.

Distribution: Papua New Guinea.

Etymology: The genus is named in honor of Dr Lesley R. Smales in recognition of her significant contribution to knowledge on the Australasian helminths.

Definition: Heligmonellidae, Nippostrongylinae. Synlophe with 14–17 ridges in both sexes. Ridges continuous. Careen absent. Ridges unequal in size, median to small. Left ridge distinct from ridge 1’. Ridge 1’, right-dorsal ridges (except right ridge) and ventral-left-ventral ridges largest. Axis of orientation oblique. Characteristic bursal pattern of type 1–4 with short common trunk. Dorsal ray divided within proximal half. Each spicule ending in one tip. SpL/BL: 7%.

### VI- Genus *Sanduanensis* n. gen. (Fig. 2F)

urn:lsid:zoobank.org:act:0CAA299C-8055-4D0D-8423-B6F482E02B02

Type and sole species: *Sanduanensis dividua* (Smales, 2014) n. comb.

Hosts: Muridae (Rodentia).

Host site: Small intestine.

Distribution: Papua New Guinea.

Etymology: The genus is named after one of the localities where the species was found.

Definition: Heligmonellidae, Nippostrongylinae. Synlophe with 16 ridges in both sexes. Dorsal ridges continuous, ventral ridges discontinuous. Careen absent. Ridges unequal in size, median to small. Left ridge distinct from ridge 1’. Mid-left and mid-right ridges largest. Absence of cuticular dilatations. Characteristic bursal pattern of type 1–4. Dorsal ray divided within proximal half. Each spicule ending in one tip. SpL/BL: 13.2%.

### VII- Genus *Parasabanema* Smales & Heinrich, 2010 (Figs. 2G and 3G)

Type species: *Parasabanema szalayi* Smales & Heinrich, 2010.

Hosts: Muridae (Rodentia).

Host site: Small intestine.

Distribution: New Guinea, Australia.

Definition: Heligmonellidae, Nippostrongylinae. Synlophe with 22–45 ridges in both sexes. Ridges continuous. Careen absent. Small ridges slightly unequal in size. Mid-left, right-right-dorsal and mid-ventral ridges largest. Right-right-ventral ridges minute. Presence of left-dorsal and right-ventral cuticular dilatations. Axis of orientation oblique. Characteristic bursal pattern of type 1–4. Dorsal ray divided within distal half. Each spicule ending in one tip. SpL/BL: 8%.

Other species: *Parasabanema praeputiale* (Gibbons & Spratt, 1995) n. comb.

### VIII- Genus *Equilophos* n. gen. (Figs. 2H and 3H)

urn:lsid:zoobank.org:act:2B27AD22-D6F8-4CFF-8C68-B9682F6E92FF

Type species: *Equilophos polyrhabdote* (Mawson, 1961) n. comb.

Hosts: Muridae (Rodentia).

Host site: Small intestine.

Distribution: Australia.

Etymology: The genus was named in this way because the ridges are of similar size.

Definition: Heligmonellidae, Nippostrongylinae. Synlophe with 35–36 ridges in both sexes. Ridges continuous. Careen absent. Ridges small, of similar size, except mid-right ridges, minute. Axis of orientation almost subfrontal. Characteristic bursal pattern of type 1–4. Each spicule ending in one tip. SpL/BL: 10–11%.

Other species: *Equilophos similis* (Smales, 2009) n. comb.

### Comments

*Odilia uromyos* is the most prevalent species present in *Uromys* spp. from Australia and Papua New Guinea [17, 27]. However, its synlophe has never been described in transverse section of the body. It was described as having up to 40 small, apparently subequal ridges in the distal part of the male and 48 in the female [17]. This number of subequal ridges may correspond either to the genus *Parasabanema* or to *Equilophos* n. gen. However, other characters such as the bursal pattern, which is apparently 2–3 [17] and the ratio SpL/BL of 16% do not correspond with any of these two genera. Based on the available data, *O. uromyos* cannot be placed in the generic arrangement proposed above, although the species is validated by a number of characters including the number of ridges, bursal pattern, and spicule shape. It is temporarily considered as a Nippostrongylinae *incertae sedis*.

*Odilia carinatae*, also parasitic in *Uromys* spp., was described from Papua New Guinea [21]. The synlophe was described and illustrated in males and females but the orientation of the ridges is not completely clear and its interpretation is difficult. In both sexes, a careen is absent, the ridges are unequal in size, the mid-right ridges are among the largest ridges, and there is a left or left-dorsal cuticular dilatation. However, in the male the cuticular dilatation is more marked and the largest ridges on the left side are the left-dorsal ridges, apparently two dorsal and two ventral to the axis of orientation, whereas in the female, the largest left ridges are the mid-left ridges, apparently all ventral to the axis of orientation. The synlophe characters of the female evoke those of the genus *Hughjonestrongylus*, with several species reported from *Melomys* spp., *Paramelomys*, and *Chiruromys* but also *Uromys* spp., all from Papua New Guinea [4, 22, 24, 26, this work]. The synlophe characters in both sexes, as well as the spicular characters of the male [21], enable us to distinguish these specimens from all the other species treated herein: however, they cannot be placed in the generic arrangement proposed above. Therefore, it would be preferable to consider these specimens as Nippostrongylinae *incertae sedis*, while awaiting improved descriptions of the synlophe in both sexes.

Two other species were reported as *Odilia* sp. 1 and *Odilia* sp. 2, parasitic in *Rattus* cf. *morotaiensis* from the Molucca Islands, Indonesia [14]. The synlophes of both species were described but not illustrated, and the data provided are insufficient to assign the species to any of the genera proposed here.

Table 1 provides a list of the species included in the former genus *Odilia*, with their new systematic position as proposed herein.

**Table 1.** List of species belonging to the former genus *Odilia* and their present systematic position.

Species and Reference(s)	Systematic position after this work
*Odilia bainae* Beveridge & Durette-Desset, 1992 [1]	*Chisholmia bainae* n. comb.
*Odilia brachybursa* (Mawson, 1961) [5–7, 17]	*Odilia brachybursa*
*Odilia carinatae* Smales, 2008 [21]	Nippostrongylinae *incertae sedis*
*Odilia dividua* Smales, 2014 [25]	*Sanduanensis dividua* n. comb.
*Odilia emanuelae* (Mawson, 1961) [5–7, 17, 20]	*Odilia emanuelae*
*Odilia implexa* Smales, 2008 [21]	*Hughjonestrongylus implexus* n. comb.
*Odilia mackerrasae* (Mawson, 1961) [5–7, 17]	*Odilia mackerrasae*
*Odilia mallomyos* Hasegawa & Syafruddin, 1994 [12]	*Hasegawanema mallomyos* n. comb.
*Odilia mamasaensis* Hasegawa et al., 1999 [16]	*Hasegawanema mamasaense* n. comb.
*Odilia mawsonae* (Durette-Desset, 1969) [5–7]	*Chisholmia mawsonae* n. comb.
*Odilia maxomyos* Hasegawa et al., 1999 [16]	*Hasegawanema maxomyos* n. comb.
*Odilia melomyos* (Mawson, 1961) [5–7, 17]	*Odilia melomyos*
*Odilia moatensis* Hasegawa et al., 1999 [16]	*Hasegawanema moatense* n. comb.
*Odilia polyrhabdote* (Mawson, 1961) [5–7, 17]	*Equilophos polyrhabdote* n. comb.
*Odilia praeputialis* Gibbons & Spratt, 1995 [11]	*Parasabanema praeputiale* n. comb.
*Odilia similis* Smales, 2009 [22]	*Equilophos similis* n. comb.
*Odilia tasmaniensis* Gibbons & Spratt, 1995 [11]	*Odilia tasmaniensis*
*Odilia uromyos* (Mawson, 1961) [5–7, 17, 27]	Nippostrongylinae *incertae sedis*
*Odilia sulawesiensis* Hasegawa et al., 1999 [16]	*Hasegawanema sulawesiense* n. comb.
*Odilia wauensis* Smales, 2010 [23]	*Lesleyella wauensis* n. comb.

Table 2 provides the list of genera of the “*Odilia*” complex, along with the species list, host spectrum, and biogeographical distribution.

**Table 2.** List of genera of the “*Odilia*” complex with the species list, host spectrum, and biogeographical distribution for each genus.

Genus	Species	Hosts	Region
*Odilia* Durette-Desset, 1973	*brachybursa*	*Melomys*	Australia
	*emanuelae*	*Rattus*	Australia
	***mackerrasae***	*Melomys, Uromys*	Australia
	*melomyos*	*Melomys, Uromys*	Australia
	*tasmaniensis*	*Rattus*	Tasmania
			
*Chisholmia* n. gen.	***bainae***	*Rattus, Pseudomys, Mastacomys*	Australia
	*mawsonae*	*Melomys*	Australia
			
*Equilophos* n. gen.	***polyrhabdote***	*Rattus*	Australia
	*similis*	*Melomys*	New Guinea (PNG)
			
*Hasegawanema* n. gen.	*mallomyos*	*Mallomys*	New Guinea (West Papua, Indonesia)
	***mamasaense***	*Maxomys*	Sulawesi (Indonesia)
	*maxomyos*	*Maxomys*	Sulawesi (Indonesia)
	*moatense*	*Maxomys*	Sulawesi (Indonesia)
	*sulawesiense*	*Rattus*	Sulawesi (Indonesia)
*Hughjonestrongylus* Digiani & Durette-Desset, 2014	*amplicaudae* *	*Paramelomys*	New Guinea (PI)
	***ennisae*** *	*Paramelomys*	New Guinea (PNG)
	*implexus*	*Uromys*	New Guinea (PNG)
	*mirzai* **	*Melomys*	New Guinea (PNG)
	*singauwaensis* *	*Melomys*	New Guinea (PNG, PI)
*Lesleyella* n. gen.	***wauensis***	*Lorentzimys*	New Guinea (PNG)
*Sanduanensis* n. gen.	***dividua***	*Pogonomys*	New Guinea (PNG)
			
*Parasabanema* Smales & Heinrich, 2010	*praeputiale*	*Zyzomys, Mesembriomys, Melomys*	Australia
	***szalayi***	*Paramelomys*	New Guinea (PNG, PI)

* Described as a species of *Paraheligmonelloides.*

** Described as a species of *Heligmonoides.*

Abbreviations: PNG: Papua New Guinea; PI: Province of Papua, Indonesia. In bold print, type species.

At this point, it is interesting to note that all native rodents of Australia and New Guinea (Sahul region) belong to the Murinae [18]. Among them, two groups are recognized. The first and most speciose is a taxonomically diverse but phylogenetically connected group of old endemics [19]. Recent studies support the monophyly of the old endemics, the result of a single colonization event 5.1–5.5 million years ago and subsequent rapid diversification within the region [19]. This was followed by multiple dispersal events between Australia and New Guinea, related to sea level fluctuations [19].

The other group, called the new endemics, is composed of native species of *Rattus*, which are thought to be recent colonists (from about 1 million years ago) with a history independent from that of the remaining Sahulian murines [19].

The new generic arrangement proposed for the nippostrongylines belonging to the “*Odilia* complex” seems to follow a distribution which is apparently more related to different geographic areas than to definite host groups (see Table 2).

Species of *Hughjonestrongylus*, *Sanduanensis*, and *Lesleyella* are distributed among several genera of hosts belonging to different groups of the old endemics, but are all restricted to New Guinea.

Species of *Parasabanema* have a Sahulian distribution, being present in Australia and New Guinea and parasitizing members of different groups of the old endemics. Species of *Equilophos* also have a Sahulian distribution, with one species in an old endemic of New Guinea and another in a native *Rattus* of Australia.

Species of *Odilia* and *Chisholmia* are found in mainland Australia (one species in Tasmania), parasitizing members of different groups of old endemics but also species of native *Rattus* (new endemics).

All these taxa of Sahulian distribution are likely to have undergone high diversification accompanying that of their hosts, diversification which followed the first colonization of Sahul by the ancestors of the old endemics. The presence of certain genera (*Equilophos*, *Parasabanema*) in both Australia and New Guinea may also reflect the latter hosts’ multiple dispersal events between these two areas. None of these genera with exclusively Sahulian distribution has species parasitic only in the new endemics. This may reinforce the hypothesis that they diversified mainly in the old endemics, whereas the presence of about four species in native species of *Rattus* could be attributed to events of host capture of the parasites from the first hosts in place.

On the other hand, the species grouped in *Hasegawanema* show a mainly extra Sahulian distribution (Table 2), with four out of five species found in endemic murines of Sulawesi [16], and only one species parasitic in West Papua (New Guinea) in one member of the old endemics [12]. Furthermore, species of *Hasegawanema* show certain synlophe characters, such as the left ridge being minute or replaced by a gap, which remind us strongly of species of *Syafruddinema* Digiani and Durette-Desset, 2014 [4], with two species parasitic in endemic murines of Sulawesi and a third in the Malay peninsula [4, 16]. A similar close relationship with representatives of Sundaland (Southeast Asia and islands of the Sunda shelf) has also been found in other endemic heligmonellids of Sulawesi such as *Hasanuddinia* Hasegawa & Syafruddin, 1994, with affinities with *Rattustrongylus* Ow-Yang et al., 1983 from peninsular Malaysia [13], or *Maxomystrongylus* Hasegawa & Syafruddin, 1997 which has congeners in Kalimantan (Borneo) [15, 16]. Such a close relationship between the heligmonellids of Sulawesi and Sundaland is not unexpected since the endemic murid fauna of Sulawesi is most closely associated with that of the Asian mainland [16, 18].

## Key to the proposed genera

1- Careen present (Figs. 2A, B).................................................................................................................................2

1’- Careen absent (Figs. 2C–H).................................................................................................................................3

2- Ridge 1’ is left ridge (Fig. 2A).

Parasites of *Melomys, Rattus, Uromys* from mainland Australia and Tasmania..................*Odilia* Durette-Desset, 1973

2’- Ridge 1’ distinct from left ridge. Left ridge minute or replaced by a gap (Fig. 2B).

Parasites of *Mallomys, Maxomys, Rattus* from Indonesia...................................................*Hasegawanema* n. gen.

3- Ridges subequal in size (Fig. 2H).

Parasites of *Rattus*, *Uromys* from Australia, *Melomys* from Papua New Guinea........................*Equilophos* n. gen.

3’- Ridges unequal in size.........................................................................................................................................4

4- Left ridges oriented perpendicularly to body surface (Fig. 3G).

Parasites of *Zyzomys, Mesembriomys, Melomys, Rattus, Uromys* from Australia..............................................................*Parasabanema* Smales & Heinrich, 2010

4’- Left ridges not oriented perpendicularly to body surface ...................................................................................5

5- Ventral-left-ventral ridges largest (Figs. 2D, E)....................................................................................................6

5’- Lateral ridges largest (Fig. 2C)............................................................................................................................7

6- Right ridge larger than left ridge (Fig. 2D).

Parasites of *Rattus, Pseudomys, Mastacomys, Melomys* from mainland Australia and Tasmania.......................................................................................................................................*Chisholmia* n. gen.

6’ Left ridge and right ridge of similar size (Fig. 2E).

Parasites of *Lorentzimys* from Papua New Guinea.........................................................................*Lesleyella* n. gen.

7- Ridges markedly unequal in size (Fig. 2C). All ridges continuous.

Parasites of *Uromys* from New Guinea..........................................................................*Hughjonestrongylus* n. gen.

7’- Ridges slightly unequal in size (Fig. 2F). Dorsal ridges continuous, ventral discontinuous.

Parasites of *Pogonomys* from New Guinea...............................................................................*Sanduanensis* n. gen.
